# Reducing Sodium Intake in Community Meals Programs: Evaluation of the Sodium Reduction in Communities Program, Arkansas, 2016–2019

**DOI:** 10.5888/pcd18.210028

**Published:** 2021-06-24

**Authors:** Christopher R. Long, Marissa J. Spear, Cari A. Bogulski, Brett Rowland, Krista Langston, Bonnie Faitak, Karra Sparks, Pearl A. McElfish

**Affiliations:** 1 College of Medicine, University of Arkansas for Medical Sciences Northwest, Fayetteville, Arkansas; 2 Office of Community Health and Research, University of Arkansas for Medical Sciences Northwest, Fayetteville, Arkansas

## Abstract

The Sodium Reduction in Communities Program (SRCP) aims to reduce dietary sodium intake through policy, systems, and environmental approaches. We evaluated progress of 3 years of SRCP activities in 3 community meals programs in northwest Arkansas. These activities sought to reduce dietary sodium intake through implementation of 1) food service guidelines, 2) procurement practices, 3) food preparation practices, and 4) environmental strategies. Mean reductions of 579 mg (−40%) in sodium served per diner and 525 mg (−22%) in sodium per 1,000 kcal served per diner were found from baseline to Year 1. Mean reductions of 499 mg (−35%) in sodium served per diner and 372 mg (−16%) in sodium per 1,000 kcal served per diner were sustained from baseline to Year 3. These results highlight the effectiveness and sustainability of sodium reduction interventions in community meals programs, whose diners experience food insecurity, have low incomes, and are at high risk for hypertension.

**Summary**

**What is known on this topic?**

High sodium intake is associated with hypertension and increased risk for cardiovascular disease, a leading cause of death for men and women in the United States.

**What does this report add?**

We evaluated a sodium-reduction intervention in community meals programs in northwest Arkansas and found substantial reductions in sodium served to diners after 3 years.

**What are the implications for public health practice?**

Sodium-reduction interventions in community meals programs, whose diners experience food insecurity, have low incomes, and are at high risk for hypertension, are effective and sustainable.

## Introduction

The 2020–2025 *Dietary Guidelines for Americans* identify the daily recommended limit for sodium intake as 2,300 mg for people aged 14 years or older (1). Adults in the US consume a mean of 3,499 mg of sodium daily (2). High sodium intake is associated with hypertension and increased risk for cardiovascular disease (3–5), which is a leading cause of death for men and women in the US (6). Evidence demonstrates that lowering excessive sodium intake decreases hypertension (4,5) and is associated with lower morbidity and mortality rates from cardiovascular diseases (3–5).

The Centers for Disease Control and Prevention (CDC) launched the Sodium Reduction in Communities Program (SRCP) in 2010 with a goal to reduce sodium intake in US populations through policy, systems, and environmental approaches to increase access to and availability of lower-sodium products (7). Program sites provide help implementing sodium reduction strategies in food service venues that serve large populations, such as hospitals, worksites, schools, early care and education centers, and higher learning institutions. Each program site evaluates the outcomes in its venues.

The University of Arkansas for Medical Sciences (UAMS) was awarded a 5-year SRCP project in 2016 to implement sodium reduction strategies in several venues in northwest Arkansas, including community meals programs (ie, programs that offer free meals to low-income clients). These venues were selected because they serve northwest Arkansas communities at heightened risk for hypertension, particularly Marshallese and Hispanic or Latino populations experiencing low incomes and food insecurity (8–10).

UAMS engaged local stakeholders from Marshallese and Hispanic or Latino communities and from the local food system (ie, food vendors, community groups, and a culinary arts school) to determine which communities would be best served by applying for an SRCP award. These meetings clarified local SRCP priorities (eg, improving access to healthy foods for populations experiencing low incomes and food insecurity) and identified potential venues (eg, community meals). Engagement of the stakeholder group has been discussed previously (10,11).

Three community meals programs implemented the sodium reduction intervention and were the focus of this study. Each program served free midday meals for onsite consumption from 1 to 4 days per week. The programs served clients who experience challenges associated with food insecurity, housing insecurity, poverty, and unemployment (10). At baseline, each program served a mean of 235 meals per meal service. Combined, the 3 programs served a mean of 103,000 meals per year during the evaluation period.

## Purpose and Objectives

In our initial evaluation from baseline to Year 1 follow-up, community meals programs were combined with foods from weekend backpack nutrition programs intended for children. In that study, which provided an overview of initial activities and effectiveness across all venues (ie, schools, weekend backpack nutrition programs, and community meals), the combined programs reduced the mean sodium served per diner by 16.6% (10). The prior study provided an overview of initial activities and effectiveness across all venues (ie, schools, weekend backpack nutrition programs, and community meals). To examine the effects of sodium reduction strategies applied to a specific venue over time, this study was restricted to community meals and includes a second and third year of follow-up. Study aims were to evaluate initial sodium reduction for the community meals programs from baseline to Year 1 and to investigate the extent to which reductions were sustained in Years 2 and 3.

## Intervention Approach

The intervention approach included implementation of 4 broad strategies recommended by SRCP: 1) food service guidelines that discuss sodium, 2) procurement practices to reduce sodium content in food purchased, 3) food preparation practices to reduce sodium content, and 4) environmental strategies to encourage reduced sodium intake (eg, moving salt shakers from dining tables to the periphery of the dining area) (Table 1).

**Table 1:** Sodium Reduction Intervention Activities Implemented by 3 Community Meals Programs Participating in the Sodium Reduction in Communities Program, Arkansas, 2016–2019

Intervention Strategy	Activities to Address Each Strategy
Food service guidelines that discuss sodium	• Implemented comprehensive food service guidelines that include sodium reduction standards and practices. Example: Adjusted donation requests to specify low- and lower-sodium products (eg, low-sodium or reduced-sodium canned corn)
Procurement practices to reduce sodium content	• Implemented standardized purchasing lists with lower sodium items.^a^ Example: Switched from purchasing canned to frozen vegetables (eg, frozen green beans). • Participated in taste tests of lower-sodium ingredients for program staff. Example: Conducted taste tests with lower-sodium Thai chili sauce for grain bowls.
Food preparation practices to reduce sodium content of menu items and meals	• Implemented policy to eliminate “free salting.” Example: Implemented policy for chefs to follow recipes for measuring salt rather than adding salt to taste. • Developed and served recipes for lower-sodium menu items that incorporate donated foods. Example: Incorporated donated spinach into lasagna roll-up recipe. • Implemented rinsing of canned vegetables to reduce sodium content. Example: Encouraged chefs to rinse canned vegetables (eg, black beans) with water.
Environmental strategies that encourage reductions in dietary sodium intake	• Placed posters featuring sodium-reduction messages in food preparation areas. Example: “Shake the Habit” poster depicting spices with a message that reads “Shake the Salt Habit, Spice it Up!” • Placed multilingual educational signs and dining table tents that address sodium reduction in dining areas. Example: “Eat More Color” table tents depicting tips for adding fruits and vegetables to meals in English, Spanish, and Marshallese. • Received monthly newsletters of sodium-reduction tips sent by UAMS staff. Example: Suggested using onions, garlic, and vinegars in place of salt to add flavor to foods. • Moved salt shakers away from dining tables to locations across the room. Example: Replaced salt shakers on dining tables with black pepper shakers.

Abbreviations: UAMS, University of Arkansas for Medical Sciences.

a All 3 programs implemented each of the activities in Year 1 and sustained them throughout the evaluation period, with the exception of standardized purchasing lists, which were not implemented until Year 2.

Representatives from each community meals program met 9 to 12 times per year from Years 1 to 3 with the UAMS team and participated in annual peer learning–exchange trainings in Years 1 to 3. The trainings were, in some instances, presented in collaboration with the Brightwater Center for the Study of Food or a University of Arkansas Culinary Nutrition instructor. Trainings often involved food preparation demonstrations (eg, knife skills training, fruit and vegetable preparation), lower-sodium product taste-testing, and feedback sharing between UAMS staff and community meals program staff.

All 3 programs implemented activities across the 4 strategies in Year 1; however, none of the programs implemented standardized purchasing lists (eg, commitments to prioritizing low-sodium or “no added salt” items when available from vendors) until Year 2. By Year 2, all 3 programs implemented all of the activities across the 4 strategies. Annually, UAMS staff supported each program’s staff to develop a comprehensive work plan to ensure sustainment of the strategies. For example, a UAMS registered dietitian continuously collaborated with community meals staff to create lower-sodium recipes by incorporating food items commonly donated by restaurants and grocery retailers (eg, adding low-fat milk or yogurt to donated salad dressings to lower sodium). Beginning in Year 2, UAMS’s registered dietitian worked with community meals staff to ensure reductions in sodium did not compromise energy intake for the programs’ food insecure diners. Each site sustained each activity through Year 3 and beyond.

## Evaluation Methods

Baseline data from the 3 community meals programs were collected between November 2016 and February 2017, before intervention implementation. Beginning in fall 2017, follow-up data were collected annually in October for Years 1, 2, and 3, attempting to minimize variability due to seasonal factors. At each program, baseline data were collected for all community meals served within a 4-week period, which included 4 to 12 meal services per program, depending on meal service frequency. At each program, annual follow-up data were collected for all community meals served within a 2- to 4-week period, which included 4 to 6 meal services per program, depending on meal service frequency. For each of the evaluated meals, data collected included the name, ingredients, and serving size of each menu item offered; the numbers of diners and of each menu item served; and sodium, energy, and other nutritional content of all food items distributed. Evaluators observed menus, ingredients, and serving sizes. Program staff provided numbers of diners and food items served.

These programs did not provide diners a choice of meals or serving sizes. In each program at any given meal service, every diner was served the same food items and the same serving sizes. For this reason, within each program, sodium served per diner on a given day was equivalent to the milligrams of sodium per meal offered on that day. For each annual evaluation period, mean sodium served per diner was calculated for each program. This calculation was a weighted mean of each evaluated day’s sodium served per diner weighted by the number of diners served on that day.

To evaluate potential unintended consequences of the sodium reduction strategies on energy content, means of energy served per diner were calculated for each program. These quantities were calculated similarly to the means for sodium content. To evaluate the changes in sodium served relative to the changes in energy, the mean number of milligrams of sodium per 1,000 kcal served per diner was calculated for each program. First, the mean number of milligrams of sodium per 1,000 kcal for each meal was calculated by computing the quotient of the milligrams of sodium served per diner divided by the calories served per diner and then multiplying the quotient by 1,000. This number was then multiplied by the number of diners served that meal. Next, this weighted number of milligrams of sodium per 1,000 kcal ratio was then summed across all of that program’s meals in each data collection period and divided by the total number of diners served by that program across all meals in that data collection period.

Nutritional content was obtained from Nutrition Facts labels or from the Nutritionist Pro database (Axxya Systems, LLC). Nutritional content across all 4 data collection periods was calculated using Excel 2013 (Microsoft Corp), R (version 3.5.2; R Foundation for Statistical Computing), and RStudio (version 1.1.463; RStudio, Inc). This evaluation was determined to be exempt by the UAMS institutional review board.

## Results

Across the 3 programs, the mean amount of sodium served per diner from baseline to Year 1 follow-up decreased from 1,443 mg to 864 mg (−40%). The mean amount of sodium served per diner in Year 2 follow-up was 920 mg, which was more than the 864 mg observed in Year 1 follow-up (+6%) but less than baseline (−36%). In Year 3 follow-up, the mean amount of sodium served per diner was 944 mg, which was more than Year 2 but less than baseline (−35%) (Table 2).

**Table 2:** Mean Diners, Energy, and Sodium Content, From Baseline Through Year 3, at 3 Community Meals Programs Participating in the Sodium Reduction in Communities Program, Arkansas, 2016–2019^a^

Program/Variables	Baseline	Year 1	Year 2	Year 3
**Program A**				
Diners per meal service, n	261	246	273	292
Energy per diner, kcal	541	478	649	349
Sodium per diner, mg	1,403	1,067	1,050	748
Sodium per 1,000 kcal per diner, mg	2,661	2,220	1,665	2,151
**Program B**				
Diners per meal service, n	220	185	253	297
Energy per diner, kcal	691	311	517	507
Sodium per diner, mg	1,310	313	712	905
Sodium per 1,000 kcal per diner, mg	1,962	1,094	1,345	1,837
**Program C**				
Diners per meal service, n	202	195	225	270
Energy per diner, kcal	704	609	588	643
Sodium per diner, mg	2,034	1,262	1,033	1,329
Sodium per 1,000 kcal per diner, mg	2,798	2,323	1,779	2,131
**Overall^b^**				
Diners per meal service, n	235	210	253	288
Energy per diner, kcal	621	453	586	479
Sodium per diner, mg	1,443	864	920	944
Sodium per 1,000 kcal per diner, mg	2,397	1,872	1,571	2,025

a Data were collected at each program immediately before intervention implementation and again in September or October for Years 1–3. Baseline data were collected during 4–12 consecutive days of service per program, and annual follow-up data were collected during 4–6 consecutive days of service per program. At baseline, none of the intervention activities had been implemented at any of the programs.

b Overall represents the combined data from Programs A, B, and C in each year.

The mean energy served per diner from baseline to Year 1 follow-up decreased from 621 kcal to 453 kcal (−27%). The mean energy served per diner in Year 2 follow-up was 586 kcal, which is more than the 453 kcal observed in Year 1 follow-up (+29%) but less than baseline (−6%). The mean energy served per diner in Year 3 follow-up was 479 kcal, which is less than Year 2 follow-up (−18%) and less than baseline (−23%) (Table 2).

The mean number of milligrams of sodium per 1,000 kcal served per diner from baseline to Year 1 follow-up decreased from 2,397 mg to 1,872 mg (−22%). The mean number of milligrams of sodium per 1,000 kcal served per diner in Year 2 follow-up was 1,571 mg, which is less than the 1,872 mg observed in Year 1 follow-up (−16%) and less than baseline (−34%). The mean number of milligrams of sodium per 1,000 kcal served per diner in Year 3 follow-up was 2,025 mg, which is more than Year 2 follow-up (+29%) but less than baseline (−16%) (Table 2).

Among the 3 programs, Program B demonstrated a noticeable reduction in amount of sodium served per diner from baseline to Year 1 from 1,310 mg to 313 mg (−76%). This reduction in sodium co-occurred with a reduction in mean energy served per diner from baseline to Year 1 follow-up from 691 kcal to 311 kcal (−55%). The amount of energy served per diner increased from Year 1 to Year 2 from 311 kcal to 517 kcal (+66%). The amount of energy served per diner then remained similar from Year 2 to Year 3, with Year 3 at 507 kcal (−2%). Mean number of milligrams of sodium per 1,000 kcal served per diner by Program B decreased from baseline to Year 1 (−44%) and then moved closer to baseline in Year 2 (−31% relative to baseline) and Year 3 (−6% relative to baseline).

## Implications for Public Health

The northwest Arkansas SRCP intervention in community meals programs reduced sodium served per diner and per meal and sustained reductions from Years 1 to 3. These results highlight the effectiveness and sustainability of sodium reduction interventions in community meals programs. The 3 community meals programs in this study ended Year 3 serving 944 mg of sodium per diner, which exceeds the 800 mg per meal recommended by CDC’s *Smart Food Choices* guidelines for public facilities (12). The 3 programs ended Year 3 serving 2,025 mg of sodium per 1,000 kcal served, which exceeds the chronic disease risk reduction levels for sodium indicated in 2020–2025 *Dietary Guidelines for Americans* (ie, 2,300 mg of sodium per day for people aged 14 years or older) (1).

However, between baseline and Year 3, sodium served per diner dropped by 499 mg of sodium and number of milligrams of sodium per 1,000 kcal dropped by 372 mg. Daily sodium reductions of this magnitude achieved at the national level would result in significant savings in health care costs and significant gains in national productivity (13,14). Moreover, sodium reductions in community meals programs, many of whose diners face food insecurity, low incomes, and high risk for hypertension, may be particularly effective (8,9).

A key finding of this study is that sodium reduction was sustained throughout the evaluation period. In this study, levels of sodium served decreased sharply from baseline to Year 1 but began trending back toward baseline between Years 1 and 3. Challenges to sustaining the initial sodium reduction included turnover in staff at the meal programs and the gradual adjustments in the amount of energy served to mitigate the sharp drop in mean calories served per diner between baseline and Year 1. To sustain a meaningful reduction from baseline through Year 3, this intervention relied on durable policy, systems, and environment changes implemented during Years 1 and 2. Sustainability was further enhanced by evaluation efforts focused on process improvement. Each year, UAMS staff partnered with staff at each meal program to use evaluation results from their program to target the prior year’s high-sodium items. This approach facilitated efficient use of program staff time to deploy new procurement and food preparation strategies to address the highest sodium items. In most cases, these strategies involved changes to recipes or ingredients rather than elimination of menu items.

One challenge of this intervention’s process-focused evaluation approach is the time- and staff-intensive nature of data collection and analyses. This approach relied on technical expertise from registered dietitians, data collectors, and other UAMS staff, as well as close coordination with meal program staff. However, by assuming much of the evaluation effort, UAMS empowered meal program staff to focus their intervention-related effort on collaborating to develop strategies to address high-sodium items identified by the evaluation. The process-focused evaluation approach allowed UAMS and meal program staff to identify and address unintended consequences of the intervention, such as the Year 1 reduction of calories in meals served to food-insecure diners.

A limitation of this evaluation approach is that its time-intensive nature precluded data collection from nonintervention meal programs to use as comparison sites. Similarly, to conserve evaluator time and effort, the evaluation focused on sodium served rather than sodium consumed, and it did not incorporate consideration of food waste. Another limitation relates to the evaluation’s attempt to minimize effects of seasonal variation by collecting each year’s follow-up data in October. Although these 3 programs relied heavily on canned fruits and vegetables throughout the year, seasonal variations in availability of fresh foods may have resulted in differences in sodium served in community meals programs during the year. However, our findings build on evidence established by SRCP in other venues (15), reinforcing evidence of these interventions’ effectiveness in reducing sodium across venues. Our study adds to the evidence base by showing that reductions in sodium served in community meals programs were sustained from Years 1 to 3. Ongoing evaluation of Years 4 and 5 will demonstrate the extent to which the reduction in sodium intake in community meals will be further sustained.

## References

[1] US Department of Agriculture, US Department of Health and Human Services. Dietary guidelines for Americans, 2020–2025. https://www.dietaryguidelines.gov/. Accessed May 3, 2021.

[2] Wallace TC , Cowan AE , Bailey RL . Current sodium intakes in the United States and the modelling of glutamate’s incorporation into select savory products. Nutrients 2019;11(11):E2691. 10.3390/nu11112691 31703311 PMC6893472

[3] Grillo A , Salvi L , Coruzzi P , Salvi P , Parati G . Sodium intake and hypertension. Nutrients 2019;11(9):E1970. 10.3390/nu11091970 31438636 PMC6770596

[4] Cook NR , Appel LJ , Whelton PK . Sodium intake and all-cause mortality over 20 years in the trials of hypertension prevention. J Am Coll Cardiol 2016;68(15):1609–17. 10.1016/j.jacc.2016.07.745 27712772 PMC5098805

[5] Cook NR , Appel LJ , Whelton PK . Lower levels of sodium intake and reduced cardiovascular risk. Circulation 2014;129(9):981–9. 10.1161/CIRCULATIONAHA.113.006032 24415713 PMC4181831

[6] Heron M . Deaths: leading causes for 2017. Natl Vital Stat Rep 2019;68(6):1–77. 32501203

[7] Centers for Disease Control and Prevention, US Department of Health and Human Services. About the Sodium Reduction in Communities Program. https://www.cdc.gov/dhdsp/programs/about_srcp.htm. Accessed January 2, 2020.

[8] Villarroel M , Blackwell D , Jen A . Tables of summary health statistics for US adults: 2018 National Health Interview Survey. http://www.cdc.gov/nchs/nhis/SHS/tables.htm. Accessed May 3, 2021.

[9] Gregory CA , Coleman-Jensen A ; US Department of Agriculture. Food insecurity, chronic disease, and health among working-age adults. 2017. https://www.ers.usda.gov/webdocs/publications/84467/err-235.pdf. Accessed November 29, 2018.

[10] Long CR , Rowland B , Langston K , Faitak B , Sparks K , Rowe V , Reducing the intake of sodium in community settings: evaluation of year one activities in the Sodium Reduction in Communities Program, Arkansas, 2016–2017. Prev Chronic Dis 2018;15:180310. 10.5888/pcd15.180310 30576274 PMC6307830

[11] McElfish PA , Kohler P , Smith C , Warmack S , Buron B , Hudson J , Community-driven research agenda to reduce health disparities. Clin Transl Sci 2015;8(6):690–5. 10.1111/cts.12350 26573096 PMC4703475

[12] Centers for Disease Control and Prevention. Smart food choices: how to implement food service guidelines in public facilities, 2018. https://www.cdc.gov/obesity/downloads/strategies/Smart-Food-Choices-508.pdf. Accessed May 3, 2021.

[13] Dall TM , Fulgoni VL 3d , Zhang Y , Reimers KJ , Packard PT , Astwood JD . Potential health benefits and medical cost savings from calorie, sodium, and saturated fat reductions in the American diet. Am J Health Promot 2009;23(6):412–22. 10.4278/ajhp.080930-QUAN-226 19601481

[14] Dall TM , Fulgoni VL 3d , Zhang Y , Reimers KJ , Packard PT , Astwood JD . Predicted national productivity implications of calorie and sodium reductions in the American diet. Am J Health Promot 2009;23(6):423–30. 10.4278/ajhp.081010-QUAN-227 19601482

[15] Jordan J , Hickner H , Whitehill J , Yarnoff B . CDC’s Sodium Reduction in Communities Program: evaluating differential effects in food service settings, 2013–2016. Prev Chronic Dis 2020;17:190446. 10.5888/pcd17.190446 32730201 PMC7417026

